# PCR-Based Screening for *mcr*-7, *mcr*-8, and *mcr*-9 in *Acinetobacter baumannii–Acinetobacter calcoaceticus* Complex Isolates from Veterinary and Environmental Sources

**DOI:** 10.3390/antibiotics15101040

**Published:** 2026-10-21

**Authors:** Thérèsse C. N. Holmström, Luana O. Silva, Sophia M. P. O. Costa, Leticia B. Pinto, Mário T. Makita, Cláudio M. Rocha-de-Souza, Shana de M. O. Coelho, Dayanne A. de Melo, Irene S. Coelho, Miliane M. S. de Souza

**Affiliations:** 1 Department of Veterinary Microbiology and Immunology, Federal Rural University of Rio de Janeiro (UFRRJ), BR-465, Km 7, Seropédica 23897-000, RJ, Brazil; luana.oliveira2001@outlook.com (L.O.S.); sophiapotz@ufrrj.br (S.M.P.O.C.); lebp.bio@hotmail.com (L.B.P.); tatsuomakita@gmail.com (M.T.M.); shana@ufrrj.br (S.d.M.O.C.); irenecs@yahoo.com (I.S.C.); milianemss@gmail.com (M.M.S.d.S.); 2 Laboratory of Research on Hospital Infection, Oswaldo Cruz Institute (Fiocruz), Avenida Brasil, 4365, Manguinhos, Rio de Janeiro 21040-900, RJ, Brazil; claudio.rocha@ioc.fiocruz.br; 3 Department of Medicine, University of Vassouras, Avenida Expedicionário Oswaldo de Almeida Ramos, 280, Centro, Vassouras 27700-000, RJ, Brazil; daymelo.com@gmail.com

**Keywords:** antimicrobial resistance, *Acb* complex, colistin, *mcr* genes, One Health, Brazil

## Abstract

Background/Objectives: The *Acinetobacter baumannii–calcoaceticus* (*Acb*) complex comprises opportunistic pathogens of increasing relevance in human and veterinary medicine due to their capacity to acquire multidrug resistance. colistin, a last-resort antimicrobial for carbapenem-resistant *A. baumannii* (CRAB), is increasingly challenged by the emergence of resistance determinants, including those encoded by the mobilized colistin resistance (*mcr*) gene family. This study aimed to screen for *mcr* genes in *Acb* complex isolates recovered from animal and environmental sources in Rio de Janeiro State, Brazil. Methods: Thirty-five isolates obtained from dogs, cats, horses, and soil between 2018 and 2020 were genotypically identified using multiplex PCR. Of these, 23 successfully recovered isolates were screened for *mcr* variants (*mcr-1* to *mcr-9*) by multiplex PCR. Results: Five isolates (21.7%) yielded amplicons of expected sizes consistent with the presence of *mcr*-associated determinants, including two consistent with *mcr*-7, one with *mcr*-8, and two with *mcr*-9. Because amplicon sequencing and phenotypic colistin susceptibility testing were not performed, these findings should be interpreted as preliminary molecular screening results. Conclusions: These findings indicate the presence of PCR-detectable *mcr*-associated genetic targets in isolates from animal and environmental sources, highlighting the need for further molecular characterization and integrated surveillance under a One Health framework.

## 1. Introduction

The genus *Acinetobacter* comprises ubiquitous, Gram-negative, non-fermentative bacilli that have been increasingly recognized as opportunistic pathogens in both human and veterinary medicine. Within this genus, the *Acinetobacter baumannii–Acinetobacter calcoaceticus* (*Acb*) complex—encompassing *A. baumannii*, *A. calcoaceticus*, *A. pittii*, and *A. nosocomialis*—has gained particular notoriety due to its persistence in healthcare environments and its remarkable ability to acquire multidrug resistance [1,2]. The World Health Organization (WHO) has classified *A. baumannii* as a “critical priority pathogen,” underscoring the urgent need for new therapeutic and surveillance strategies to control infections caused by carbapenem-resistant *A. baumannii* (CRAB) [3].

Carbapenems, considered last-resort antimicrobials for the treatment of infections caused by multidrug-resistant Gram-negative bacteria, remain essential due to their broad-spectrum activity and clinical efficacy. However, *A. baumannii* exhibits an extraordinary capacity to persist on both abiotic and biotic surfaces, facilitating nosocomial transmission and contributing to prolonged outbreaks [4,5]. Consequently, the management of CRAB infections represents a major clinical challenge, particularly in intensive care units (ICUs), where critically ill patients are at increased risk. Recent therapeutic guidelines recommend empirical combination regimens, frequently incorporating colistin (polymyxin E) as a key component [6].

Colistin, a cyclic polypeptide antibiotic introduced in the 1960s, was largely abandoned due to its dose-dependent nephrotoxicity [7,8]. However, with the global rise in multidrug-resistant infections, it has re-emerged as one of the few remaining therapeutic options for CRAB infections [9].

Historically, resistance to colistin was attributed exclusively to chromosomal mutations, limiting its transmission to vertical inheritance. However, the widespread and often indiscriminate use of colistin has driven the emergence of plasmid-mediated resistance mechanisms, enabling horizontal gene transfer across diverse bacterial species [10]. In 2016, the first mobilized colistin resistance gene (*mcr*-1) capable of plasmid-mediated transfer was identified [11]. Since then, multiple *mcr* families and variants have been described in bacterial isolates from human, animal, and environmental sources across more than sixty countries [12].

Although the dissemination of *mcr* genes has been extensively documented in members of the Enterobacteriaceae family, their occurrence in the *Acb* complex remains relatively rare, particularly in isolates of animal origin. Considering the growing evidence that animals may act as reservoirs and disseminators of antimicrobial resistance determinants, the detection of mobile *mcr*-associated colistin resistance determinants in *Acb* complex species from animal sources is of significant concern.

Therefore, the present study aimed to screen *Acb* complex isolates obtained from veterinary and environmental sources for *mcr* genes using multiplex PCR. To our knowledge, this is the first report of PCR-based detection of *mcr*-7, *mcr*-8, and *mcr*-9 in veterinary *Acinetobacter* isolates from the state of Rio de Janeiro, Brazil. These findings reinforce the importance of continuous antimicrobial resistance surveillance in veterinary medicine and highlight the need for a One Health approach to monitor the dissemination of resistance across human, animal, and environmental interfaces.

## 2. Results

Among the 180 non-fermenting Gram-negative coccobacilli (NFGNC) isolates analyzed, 35 isolates were selected for further identification and characterization as *Acinetobacter* spp. Proteomic identification by MALDI-TOF MS provided further taxonomic resolution of these isolates. Among the 35 isolates, 14/35 (40.00%) were identified as *A. baumannii*, 11/35 (31.43%) as *A. pittii*, 4/35 (11.43%) as *A. nosocomialis*, 3/35 (8.57%) as non-*Acb* complex species, comprising two *A. ursingii* and one *A. venetianus*, 1/35 (2.86%) was identified only at the genus level (*Acinetobacter* spp.), and 2/35 (5.71%) could not be identified by MALDI-TOF MS.

For molecular confirmation of the *Acb* complex species, a multiplex PCR assay targeting the *recA* gene (*Acinetobacter* spp.), *gyrB* gene (*A. baumannii* and *A. nosocomialis*), and species-specific internal transcribed spacer (ITS) regions for *A. baumannii* and *A. pittii* was performed according to Chen et al. [13]. Molecular identification by multiplex PCR classified 14/35 (40.00%) isolates as *A. pittii*, 13/35 (37.14%) as *A. baumannii*, 6/35 (17.14%) as *A. nosocomialis*, and 2/35 (5.71%) as *Acinetobacter* spp.

Overall, agreement between MALDI-TOF MS and multiplex PCR was observed for most isolates, particularly those identified as *A. baumannii* and *A. pittii*. However, discrepancies were observed for several isolates. Two isolates identified as *A. ursingii* and one as *A. venetianus* by MALDI-TOF MS were assigned to species within the *Acb* complex by multiplex PCR. In addition, some isolates identified as *A. nosocomialis* by MALDI-TOF MS were classified as *A. pittii* or *Acinetobacter* spp. by multiplex PCR, while isolates that were not identified by MALDI-TOF MS were assigned to *A. nosocomialis* or *A. pittii* by the molecular assay. These discrepancies highlight the challenges associated with species-level identification within the *Acb* complex and the limitations of individual identification methods.

The isolates were recovered from diverse anatomical and environmental sources, with urine being the most frequent sample type, followed by skin, otological, nasal, oral, and soil samples (Table 1).

Overall agreement between MALDI-TOF MS and multiplex PCR was assessed using Cohen’s kappa coefficient. The analysis showed good agreement between the two methods (κ = 0.729; 95% CI: 0.509–0.949; *p* = 8.29 × 10^−11^) (Table 2). The overall percentage agreement was also calculated as a complementary measure of concordance. The overall percentage agreement between MALDI-TOF MS and multiplex PCR was 80.0% (28/35).

Among the 35 *Acinetobacter* spp. isolates, 33 were assigned to the *Acb* complex. Of these, 12 isolates were lost during storage or processing and could not be recovered, whereas the remaining 23 isolates were successfully recovered and further screened for *mcr* genes.

Among these, five isolates (21.7%) were PCR screening-positive, yielding amplicons consistent with at least one *mcr* target. Specifically, two isolates (8.7%) were PCR-positive for *mcr*-7, one (4.3%) for *mcr*-8, and two (8.7%) for *mcr*-9. (Table 1). Phenotypic colistin susceptibility was not assessed; therefore, the detection of PCR amplicons does not demonstrate phenotypic colistin resistance.

Notably, all *mcr*-positive isolates were recovered from urine samples of dogs, except for one environmental isolate obtained from soil, indicating that *mcr*-associated resistance determinants were detected in both animal-associated and environmental isolates within the study population. All *mcr*-positive isolates belonged to clinically relevant *Acb* complex species. Phenotypic colistin susceptibility testing was not performed in the present study. Therefore, the detection of PCR amplicons consistent with *mcr* genes should be interpreted as a molecular screening finding and does not establish phenotypic colistin resistance or reduced susceptibility.

## 3. Discussion

The molecular identification approach employed in this study presents an inherent limitation, as the multiplex PCR assay enables the differentiation of only three species within the *Acb* complex. Although this limitation does not preclude the interpretation of the present findings, it restricts the taxonomic resolution of the assay and may contribute to discrepancies when compared with other identification methods. This molecular approach was complemented by the use of MALDI-TOF MS for proteomic identification, a technique that has substantially improved routine microbiological diagnostics by reducing turnaround time, sample requirements, and costs while providing reliable species-level identification in many bacterial groups [14]. However, reliable species-level identification by MALDI-TOF MS is generally associated with confidence scores above 2.3. In the present study, although all isolates yielded scores above 2.0, none exceeded the 2.3 threshold, which may have contributed to the discrepancies observed between MALDI-TOF MS and multiplex PCR assignments. According to the interpretation of agreement coefficients proposed by Fleiss et al. [15], values above 0.75 indicate excellent agreement. According to Altman [16], coefficients ranging from 0.61 to 0.80 indicate good agreement, whereas perfect agreement is observed only when the coefficient equals 1.00. The kappa value obtained in the present study (0.729) therefore indicates good agreement between MALDI-TOF MS and multiplex PCR. Thus, despite the discrepancies observed for some isolates, both methods provided concordant species-level assignments for most *Acb* complex isolates and can be considered complementary approaches for routine identification. Therefore, MALDI-TOF MS and multiplex PCR are good alternatives for identifying isolates belonging to *Acb* complex species. Nevertheless, the development of multiplex PCR assays incorporating additional molecular targets for other *Acb* complex species could improve taxonomic resolution and provide a complementary approach to proteomic identification. Importantly, the identification of the three *Acb* complex species targeted by the multiplex PCR in samples associated with infectious processes in animals, as previously described by Chen et al. [17], reinforces the importance of appropriate laboratory investigation of these organisms in routine veterinary diagnostic settings. Most samples analyzed in the present study originated from dogs, followed by cats, horses, and soil, reflecting the composition of the study population rather than the distribution of *Acinetobacter* species among these sources. This refinement is particularly relevant in light of the detection of *mcr*-7, *mcr*-8, and *mcr*-9 genes by PCR in *Acb* complex isolates from animal and environmental sources, representing the first report of these resistance determinants in veterinary isolates from the state of Rio de Janeiro. These findings indicate the occurrence of *mcr*-associated resistance determinants beyond human clinical settings and reinforce the importance of investigating their genetic context and potential for horizontal transmission in non-human environments.

Until the mid-2010s, colistin resistance was believed to result exclusively from chromosomal mutations in genes associated with the biosynthesis and maintenance of lipopolysaccharide (LPS), which alter the outer membrane’s negative charge and consequently diminish the electrostatic affinity of colistin [12]. This mechanism has been extensively documented among Enterobacteriaceae [18]. However, the discovery of the *mcr*-1 gene in *Escherichia coli* isolates from China in 2016 marked a paradigm shift, revealing a plasmid-mediated mechanism capable of horizontal transmission [19].

To date, multiple *mcr* gene families and variants have been described, with *mcr*-1 remaining the most widely reported, with *mcr*-1 and *mcr*-4.3 being the most frequently reported [20]. All encode phosphoethanolamine transferase (pEtN) enzymes that modify lipid A in the outer membrane of Gram-negative bacteria, thereby reducing the net negative charge and neutralizing the electrostatic interactions essential for colistin activity [21]. The *mcr*-7, *mcr*-8, and *mcr*-9 genes exhibit conserved structural and functional features but differ in sequence identity and epidemiological context: *mcr*-7 was first identified in *Klebsiella pneumoniae* isolates from chickens in China [20]; *mcr*-8 in *K. pneumoniae* from pigs and humans in China [22]; and *mcr*-9 in multidrug-resistant *Salmonella enterica* serovar Typhimurium isolated from a human patient in the United States [23]. The wide geographic and taxonomic distribution of these genes underscores their substantial dissemination potential [24].

Although *mcr* genes are most frequently reported in *E. coli*, *K. pneumoniae*, and *Salmonella* spp. [25], their detection in *Acinetobacter* species remains uncommon, particularly among veterinary and environmental isolates. This apparent scarcity may reflect biological barriers to horizontal gene acquisition, differences among ecological niches, or limited surveillance in non-human settings. The detection of *mcr*-7, *mcr*-8, and *mcr*-9 by multiplex PCR in the present study is therefore noteworthy. However, given the relatively limited and geographically restricted sampling, with most isolates originating from a single veterinary diagnostic laboratory and only two isolates obtained from poultry-associated soil, these findings should be interpreted as evidence of the occurrence of these determinants among the isolates evaluated rather than as an indication of their prevalence or widespread distribution in veterinary or environmental populations. Further studies involving larger and geographically diverse collections of animal and environmental isolates are needed to determine the frequency, distribution, and epidemiological relevance of these determinants.

An important consideration is the potential genotype–phenotype discrepancy. The presence of *mcr* genes does not necessarily correlate with phenotypic resistance to colistin, as their expression and phenotypic effect may depend on regulatory elements and the genetic context in which the determinant is located. Furthermore, the multiplex PCR approach used here, while efficient for screening, does not distinguish between chromosomal and plasmid-borne locations, nor does it confirm the specific variant within each *mcr* group. Plasmid-borne *mcr* determinants may have greater potential for horizontal dissemination, whereas chromosomal localization may be associated with greater genetic stability. However, the genomic location of the determinants detected in the present study was not determined. Without whole-genome sequencing or plasmid characterization, the mobility and exact genetic context of these determinants remain uncertain.

The amplicons consistent with *mcr*-7, *mcr*-8, and *mcr*-9 in isolates recovered from canine urine and soil is noteworthy within the study population. However, these findings should not be interpreted as evidence of widespread circulation of these resistance determinants in veterinary or environmental settings, given the limited number and geographic scope of the samples analyzed. Their genetic context and mobility could not be established in the present study and require confirmation by sequencing and plasmid characterization. Broader surveillance involving isolates from multiple animal species, production systems, veterinary institutions, and environmental compartments will be necessary to assess the distribution and epidemiological significance of these determinants.

Collectively, these findings highlight the occurrence of *mcr*-associated determinants among *Acb* complex isolates from the veterinary and environmental samples evaluated and underscore the importance of integrated surveillance under a One Health framework. Given the restricted sampling design and the absence of genomic characterization, these results should be considered an initial contribution to the surveillance of *mcr* determinants in the *Acb* complex rather than evidence of their prevalence or widespread dissemination. Further studies incorporating larger and geographically diverse collections, phenotypic colistin susceptibility testing, whole-genome sequencing, and plasmid characterization are warranted to clarify the epidemiological distribution, genetic context, and potential for horizontal transmission of these determinants.

## 4. Materials and Methods

Sample collection. A total of 180 strains of non-fermenting Gram-negative coccobacilli (NFGNC) were evaluated. The isolates originated from cases of urinary tract infection, pododermatitis, otitis, and pyoderma in dogs, cats, and horses, as well as from soil samples collected in poultry environments between 2018 and 2020. Most isolates were obtained from a private veterinary diagnostic laboratory in Rio de Janeiro, while two soil isolates originated from poultry farm environments.

The strains were subcultured on MacConkey agar (MAC) (microMED, ISOFAR^®^, Duque de Caxias, RJ, Brazil) and incubated at 35 ± 2 °C for 24 h to obtain cultures of presumptive *Acinetobacter* spp. After incubation, colony morphology and lactose fermentation were evaluated by Procop [26], and non-lactose-fermenting colonies were selected. The selected strains were subsequently subjected to Gram staining and catalase, oxidase, and motility tests for preliminary phenotypic identification, following the procedures described by Procop [26].

Proteomic identification. Phenotypically characterized isolates were subjected to proteomic identification using Matrix-Assisted Laser Desorption/Ionization Time-of-Flight Mass Spectrometry (MALDI-TOF MS) (Bruker Daltonics, Bremen, Germany) at the Medical Microbiology Research Laboratory (LIMM), Federal University of Rio de Janeiro (UFRJ). Identification scores were interpreted according to manufacturer guidelines, ranging from 0.0 to 3.0: scores < 1.7 indicated no reliable identification; 1.7–1.99 suggested probable genus identification; 2.0–2.29 indicated reliable genus identification and probable species assignment; and ≥2.3 denoted reliable species-level identification. In this study, scores above 2.0 were considered acceptable for genus-level identification, and scores ≥ 2.3 were accepted as definitive at the species level.

DNA extraction by thermal lysis. Genomic DNA was extracted from cultures grown in Brain Heart Infusion (BHI) broth (Kasvi^®^, Pinhais, PR, Brazil) incubated at 35 °C for 24 h. The procedure followed the method described by Buyukcangaz et al. [27], with modifications standardized by LABACVET/UFRRJ. Briefly, bacterial pellets were resuspended in 500 µL of ultrapure water, vortexed, and subjected to heat lysis at 100 °C for 10 min. After cooling to room temperature, samples were centrifuged at 8000 rpm for 3 min. The resulting supernatant (approximately 400 µL) was transferred to new microtubes, and the pellet discarded. DNA extracts were stored at −20 °C until use.

Before performing the multiplex PCR, each primer pair was evaluated individually by simplex PCR using positive controls and a no-template control (blank) to verify amplification of the respective target. *Acinetobacter* strains with previously characterized phenotypic and/or molecular profiles were used as controls. The amplicons obtained from the positive controls were purified and sequenced to confirm the identity of the amplified targets.

The multiplex PCR assay was previously optimized at the Laboratory of Hospital Infection Research (LAPIH), Oswaldo Cruz Foundation (Fiocruz), and the optimized conditions were subsequently reproduced in the laboratory where the present study was conducted. The assay was performed using the primer sets and target regions described by Chen et al. [13]. Appropriate positive and negative controls were included in the reactions. Amplicon sizes were assessed by agarose gel electrophoresis and compared with the expected sizes for each target.

Genotypic identification of *Acb* complex species by PCR. Extracted genomic DNA was subjected to multiplex polymerase chain reaction (PCR) to confirm species-level identification within the *Acb* complex. The assay was performed using primers targeting conserved and species-specific loci, as previously described by Chen et al. [13]. The following gene targets were included: *recA* (425 bp) for the identification of the *Acinetobacter* genus; *gyrB* (294 bp) for differentiation between *A. baumannii* and *A. nosocomialis*; the internal transcribed spacer (ITS) region specific to *A. baumannii* (208 bp); and the ITS region specific to *A. pittii* (150 bp) (Table 3). PCR reactions were carried out in a final volume of 25 µL containing template DNA, reaction buffer, MgCl_2_, dNTPs, primers, and Taq DNA polymerase (Thermo Fisher Scientific, Waltham, MA, USA) under optimized cycling conditions. Amplification products were resolved by electrophoresis on 1.5% agarose gels stained with a nucleic acid dye (Thermo Fisher Scientific, Waltham, MA, USA) and visualized under ultraviolet illumination. A 100 bp DNA ladder (Thermo Fisher Scientific, Waltham, MA, USA) was used as a molecular size marker to confirm expected amplicon sizes.

Colistin resistance gene screening. The presence of *mcr* genes was investigated by multiplex PCR assays targeting *mcr* variants. The genes *mcr*-1, *mcr*-2, *mcr*-3, *mcr*-4, and *mcr*-5 were screened according to the protocol described by Rebelo et al. [28], while *mcr*-6, *mcr*-7, *mcr*-8, and *mcr*-9 were detected following the methodology proposed by Borowiak et al. [29] (Table 4). PCR amplifications were performed under previously validated conditions, and the resulting amplicons were separated by electrophoresis on agarose gels and visualized under UV light. The sizes of the PCR products were compared with expected fragment lengths to confirm the presence of each target gene. Positive controls for the respective *mcr* targets were included in each assay, and the identity of the positive-control amplicons was confirmed by sequencing. No-template controls were included in all reactions.

## 5. Conclusions

Animal and human health are intrinsically interconnected, particularly in the context of antimicrobial resistance. In this study, PCR-based detection of *mcr*-7, *mcr*-8, and *mcr*-9 in *Acb* complex isolates from veterinary and environmental sources provides, to the best of our knowledge, the first report of PCR-based screening evidence of amplicons consistent with *mcr*-7, *mcr*-8, and *mcr*-9 among *Acb* complex isolates from the state of Rio de Janeiro. These findings provide preliminary molecular evidence of the occurrence of *mcr*-associated determinants among the isolates evaluated and highlight the importance of investigating these resistance determinants in veterinary and environmental settings.

The detection of *mcr*-associated determinants in *Acb* complex isolates supports the need for further studies to determine their sequence identity, genetic context, genomic location, and potential association with phenotypic colistin resistance. Because the positive study isolates were not subjected to sequencing confirmation, colistin susceptibility testing was not performed, and plasmid characterization was not conducted, the present findings cannot establish phenotypic colistin resistance, plasmid-mediated resistance, or the potential for horizontal transmission.

Collectively, these findings provide preliminary PCR-based evidence of the occurrence of amplicons consistent with *mcr*-7, *mcr*-8, and *mcr*-9 among Acb complex isolates from veterinary and environmental sources in Rio de Janeiro State, Brazil. Given the absence of amplicon sequencing, phenotypic colistin susceptibility testing, and genomic characterization, these results should not be interpreted as evidence of confirmed *mcr* gene carriage, phenotypic colistin resistance, or horizontal transmission. Nevertheless, they reinforce the importance of incorporating Acb complex isolates from animal and environmental sources into integrated antimicrobial resistance surveillance under a One Health framework. Further studies involving larger and geographically diverse collections, sequence confirmation, colistin MIC determination, whole-genome sequencing, and plasmid characterization are warranted to clarify the epidemiological distribution, genetic context, and potential mobility of these determinants.

## Figures and Tables

**Table 1:** Origin, sampling site, identification by MALDI-TOF MS and multiplex PCR, and PCR screening results for *mcr* genes among the Acinetobacter isolates analyzed in this study.

No.	MALDI TOF MS	Multiplex PCR	Origin	Sample	*mcr*
1	*A. pittii*	*A. pittii*	Dog	urine	*mcr*-7
2	*A. pittii*	*A. pittii*	Cat	urine	
3	*A. pittii*	*A. pittii*	Dog	urine	*mcr*-9
4	*A. pittii*	*A. pittii*	Dog	urine	
5	*A. baumannii*	*A. baumannii*	Cat	skin	
6	*A. baumannii*	*A. baumannii*	Dog	skin	
7	*A. baumannii*	*A. baumannii*	Horse	skin	
8	*A. pittii*	*A. pittii*	Dog	urine	
9	*A. pittii*	*A. pittii*	Dog	urine	
10	*A. baumannii*	*A. baumannii*	Cat	urine	
11	*A. pittii*	*A. pittii*	Dog	urine	
12	*A. baumannii*	*A. baumannii*	Dog	otologic	
13	*A. pittii*	*A. pittii*	Dog	urine	
14	*A. baumannii*	*A. baumannii*	Dog	urine	*mcr*-8
15	*A. pittii*	*A. pittii*	Dog	urine	
16	*A. baumannii*	*A. baumannii*	Dog	urine	*mcr*-9
17	*A. ursingii*	*A. pittii*	Cat	urine	
18	*A. baumannii*	*A. baumannii*	Dog	skin	
19	*A. pittii*	*A. pittii*	Dog	noise	
20	*A. venetianus*	*A. nosocomialis*	Dog	skin	
21	*A. nosocomialis*	*A. nosocomialis*	Dog	otologic	
22	*A. baumannii*	*A. baumannii*	Dog	otologic	
23	*A. nosocomialis*	*Acinetobacter* spp.	Cat	urine	
24	*A. pittii*	*A. pittii*	Dog	skin	
25	*A. baumannii*	*A. baumannii*	Dog	urine	
26	*A. ursingii*	*A. nosocomialis*	Cat	noise	
27	*Acinetobacter* spp.	*Acinetobacter* spp.	Dog	urine	
28	N.I.	*A. nosocomialis*	Dog	urine	
29	N.I.	*A. nosocomialis*	Cat	otologic	
30	*A. nosocomialis*	*A. pittii*	Dog	urine	
31	*A. baumannii*	*A. baumannii*	Dog	otologic	
32	*A. baumannii*	*A. pittii*	Cat	urine	
33	*A. baumannii*	*A. baumannii*	soil		*mcr*-7
34	*A. baumannii*	*A. baumannii*	Dog	skin	
35	*A. baumannii*	*A. baumannii*	soil		

N.I. = not identified.

**Table 2:** Cohen’s kappa coefficient.

	Kappa (IC)	*p*-Value
MALDI-TOF × PCR	0.729 (0.509–0.949)	8.29 × 10^−11^

**Table 3:** Primers used in the characterization of bacterial species.

Gene/ PCR Product	Sequence (5′–3′)	Program
*recA* (425 bp)	CCTGAATCTTCTGGTAAAAC GTTTCTGGGCTGCCAAACATTAC	94 °C for 5 min; 35 cycles (94 °C for 1 min, 58 °C for 30 s and 72 °C for 30 s) and final extension of 72 °C for 10 min.
*gyrB* (294 bp)	CACGCCGTAAGAGTGCATTA AACGGAGCTTGTCAGGGTTA	
ITS region (208 bp)	CATTATCACGGTAATTAGTG AGAGCACTGTGCACTTAAG	
ITS region (150 bp)	CTCAAGAGTTTAGATTAAGCAAT GTCCGTGCGATTCTTCATCG	

**Table 4:** Colistin resistance gene screening.

Gene/ PCR Product	Sequence (5′–3′)	Program
*mcr*-1 (320 bp)	AGTCCGTTTGTTCTTGTGGC AGATCCTTGGTCTCGGCTTG	94 °C for 15 min; 25 cycles de (94 °C for 30 s, 58 °C for 1 min 30 s and 72 °C for 60 seg) and final extension of 72 °C for 10 min.
*mcr*-2 (715 bp)	CAAGTGTGTTGGTCGCAGTT TCTAGCCCGACAAGCATACC	
*mcr*-3 (929 bp)	AAATAAAAATTGTTCCGCTTATG AATGGAGATCCCCGTTTTT	
*mcr*-4 (1116 bp)	TCACTTTCATCACTGCGTTG TTGGTCCATGACTACCAATG	
*mcr*-5 (1644 bp)	ATGCGGTTGTCTGCATTTATC TCATTGTGGTTGTCCTTTTCTG	
*mcr*-6 (252 bp)	GTCCGGTCAATCCCTATCTGT ATCACGGGATTGACATAGCTAC	
*mcr*-7 (551 bp)	TGCTCAAGCCCTTCTTTTCGT TTCATCTGCGCCACCTCGT	
*mcr*-8 (856 bp)	AACCGCCAGAGCACAGAATT TTCCCCCAGCGATTCTCCAT	
*mcr*-9 (1011 bp)	GTATCCTTCCTGCCATCCTC CTTTCCATAACAGCGAGACAC	

## Data Availability

The original contributions presented in this study are included in the article. Further inquiries can be directed to the corresponding author.

## Abbreviations

The following abbreviations are used in this manuscript:

*Acb*	*Acinetobacter baumannii–calcoaceticus*
BHI	Brain Heart Infusion
bp	base pairs
CRAB	Carbapenem-resistant *Acinetobacter baumannii*
dNTPs	Deoxynucleoside Triphosphates
*gyrB*	DNA gyrase subunit B
ICUs	intensive care units
ITS	Internal Transcribed Spacer
LABACVET	Veterinary Bacteriology Laboratory
LIMM	Medical Microbiology Research Laboratory
LPS	lipopolysaccharide
MALDI-TOF MS	Matrix-Assisted Laser Desorption/Ionization Time-of-Flight Mass Spectrometry
MDR	multidrug-resistant
*mcr*	mobilized colistin resistance
NFGNC	non-fermenting Gram-negative coccobacilli
PCR	Polymerase Chain Reaction
pEtN	phosphoethanolamine transferase
*recA*	Recombinase A
Sec	seconds
UFRJ	Federal University of Rio de Janeiro
UV	Ultraviolet
WHO	World Health Organization
µL	microliter
°C	degrees Celsius
